# Pigment Diversity in Leaves of *Caladium* × *hortulanum* Birdsey and Transcriptomic and Metabolic Comparisons between Red and White Leaves

**DOI:** 10.3390/ijms25010605

**Published:** 2024-01-03

**Authors:** Yiwei Zhou, Yechun Xu, Gen-Fa Zhu, Jianjun Tan, Jingyi Lin, Lishan Huang, Yuanjun Ye, Jinmei Liu

**Affiliations:** 1 Environmental Horticulture Research Institute, Guangdong Academy of Agricultural Sciences, Guangzhou 510642, China; zhouyiwei6333@163.com (Y.Z.);; 2 Guangdong Key Laboratory of Ornamental Plant Germplasm Innovation and Utilization, Guangzhou 510642, China

**Keywords:** *Caladium* × *hortulanum*, leaf color, chlorophyll, anthocyanin, transcriptome, metabolome

## Abstract

Leaf color is a key ornamental characteristic of cultivated caladium (*Caladium* × *hortulanum* Birdsey), a plant with diverse leaf colors. However, the genetic improvement of leaf color in cultivated caladium is hindered by the limited understanding of leaf color diversity and regulation. In this study, the chlorophyll and anthocyanin content of 137 germplasm resources were measured to explore the diversity and mechanism of leaf color formation in cultivated caladium. Association analysis of EST-SSR markers and pigment traits was performed, as well as metabolomics and transcriptomics analysis of a red leaf variety and its white leaf mutant. We found significant differences in chlorophyll and anthocyanin content among different color groups of cultivated caladium, and identified three, eight, three, and seven EST-SSR loci significantly associated with chlorophyll-a, chlorophyll-b, total chlorophyll and total anthocyanins content, respectively. The results further revealed that the white leaf mutation was caused by the down-regulation of various anthocyanins (such as cyanidin-3-*O*-rutinoside, quercetin-3-*O*-glucoside, and others). This change in concentration is likely due to the down-regulation of key genes (four *PAL*, four *CHS*, six *CHI*, eight *F3H*, one *F3′H*, one *FLS*, one *LAR*, four *DFR*, one *ANS* and two *UFGT*) involved in anthocyanin biosynthesis. Concurrently, the up-regulation of certain genes (one *FLS* and one *LAR*) that divert the anthocyanin precursors to other pathways was noted. Additionally, a significant change in the expression of numerous transcription factors (12 *NAC*, 12 *bZIP*, 23 *ERF*, 23 *bHLH*, 19 *MYB*_related, etc.) was observed. These results revealed the genetic and metabolic basis of leaf color diversity and change in cultivated caladium, and provided valuable information for molecular marker-assisted selection and breeding of leaf color in this ornamental plant.

## 1. Introduction

Leaf color, a fundamental characteristic of plants, significantly contributes to the value of ornamental plants. As aesthetic preferences shift towards ornamental plants, varieties with a rich diversity of leaf colors are becoming increasingly popular and play a pivotal role in landscape greening. The types and concentrations of pigments in the leaves, such as chlorophyll, carotenoids, and anthocyanins, mainly determine the leaf color variation in ornamental plants [1]. Chlorophyll is the vital pigment for photosynthesis and the main factor influencing the greenness of plant leaves [2]. Anthocyanins are a group of water-soluble pigments, mostly located in vacuoles, that can give various colors from orange to red to blue to plant organs [3]. Exploring the molecular mechanism of leaf color diversity in ornamental plants, elucidating the different pigment metabolic pathways and regulatory networks, and providing a theoretical foundation and technical support for selecting and breeding superior varieties have significant theoretical and practical implications.

Typically, a metabolic disorder in anthocyanin results in lighter leaf color in plants. In *Acer tutcheri* Duthie, the fading of the spring red leaf is directly caused by a decrease in the anthocyanin/chlorophyll ratio, a result of the combined effect of reduced anthocyanin synthesis and increased anthocyanin degradation [4]. In *Paeonia lactiflora* Pall, researchers compared the anatomical, physiological, and molecular characteristics of leaves at the purple, purple-green, and green stages. They found that changes in surface pigment were the primary determinant of the leaf color transition from purple to green. This change was associated with the altered expression of genes involved in the synthesis and degradation of both anthocyanin and chlorophyll [5]. In white ornamental cabbage (*Brassica oleracea* var. *acephala* de Candolle), the white color of the inner leaves is attributed to an extremely low or absent level of anthocyanin biosynthesis, coupled with high chlorophyll degradation and minimal chlorophyll biosynthesis [6]. In *Phoebe bournei*, Wang et al. [7] discovered that the anthocyanin content gradually decreased as the leaves developed. They suggested that the anthocyanin cyanidin-3-*O*-glucoside and the *PbF3′H*, *PbbHLH1*, and *PbbHLH2* genes are likely to be responsible for the red leaf color. While a disorder in the anthocyanin metabolic pathway is a crucial mechanism for inducing leaf color mutation, research on the formation mechanism of red to white leaf mutation remains scarce.

Two species, *Caladium bicolor* (Ait.)Vent. and *Caladium schomburgkii* Schott., appear to be the sources of modern commercial caladium cultivars [8]. They have various leaf shapes, colors, and patterns, making them a suitable choice for potted and garden plants [9]. Previous studies have shown that single genes control the leaf shape, main vein color, leaf spot, and leaf background color of cultivated caladium [8,10,11,12,13]. The leaf color, the most attractive feature of cultivated caladium, is a key factor for plant selection. Caladium varieties display different leaf colors, such as white, red, pink, yellow, purple, and purple-black [14]. However, the genetic mechanism of leaf color diversity in cultivated caladium is poorly understood [15]. The breeding of cultivated caladium mainly depends on sexual hybridization between varieties. However, this method faces significant genetic bottlenecks due to the difficulties of interspecific hybridization and high genetic heterogeneity [8,13,16,17]. To overcome this challenge, recent studies have tried to use tissue culture to induce somatic mutation or ploidy change to create new genetic variation [18,19]. These studies have revealed that somatic mutation can result in new traits such as cultivated caladium chromosome amplification or deletion, leaf shape, and color change. However, research on the pigment diversity and molecular mechanism of leaf color change in cultivated caladium is limited, hampering further improvement of cultivated caladium leaf color.

In a previous study, 144 cultivated caladium germplasms were collected and divided into four color groups using colorimeter measurement and multivariate statistical analysis. These groups were genotyped with SSR markers to identify molecular markers significantly associated with colorimeter parameters [14]. However, the pigment composition of these four color groups remains unclear. Further analysis of the pigment differences in the leaves could enhance the understanding of the formation of cultivated caladium leaf color. In this study, the chlorophyll and anthocyanin of these four color groups were analyzed and combined with SSR markers to identify molecular markers associated with pigments. This will lay a foundation for molecular marker-assisted selective breeding of leaf color. Additionally, the red leaf ‘Lieyanxiongxin’ and its white leaf mutant were screened (Figure S1), and the cause of red to white change was analyzed by metabolomics and transcriptomics. This will provide new insights into the formation mechanism of cultivated caladium leaf color change.

## 2. Results

### 2.1. Diversity Analysis of Chlorophyll and Anthocyanin of Cultivated Caladium Germplasms

**Figure 1:**
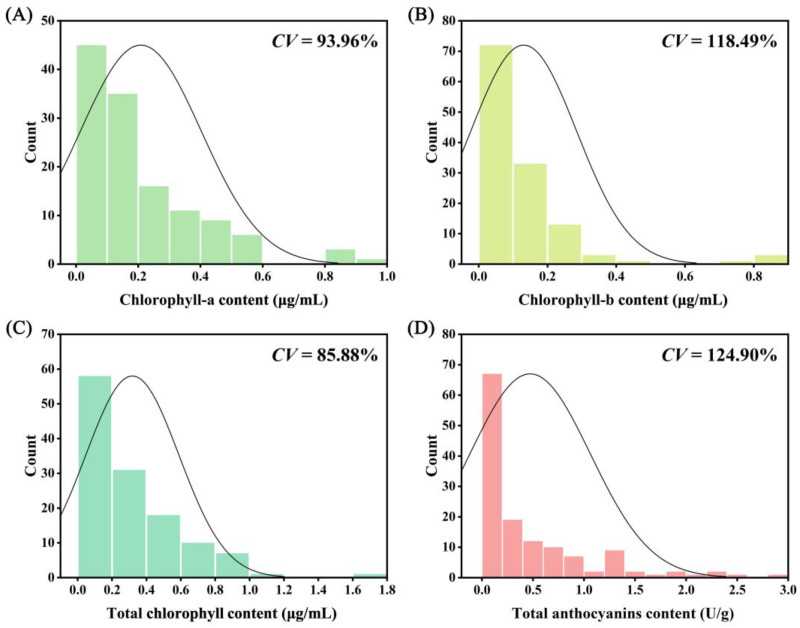
Diversity analysis of chlorophyll and anthocyanin in cultivated caladium germplasm resources. (**A**) Chlorophyll-a content. (**B**) Chlorophyll-b content. (**C**) Total chlorophyll content. (**D**) Total anthocyanins content.

Figure 1A–D illustrates the normal distribution of chlorophyll a, chlorophyll b, total chlorophyll, and total anthocyanin. These four numerical traits exhibit a continuous distribution, with coefficients of variation ranging from 85.88% to 124.90% (Tables S1 and S2). Among them, total anthocyanin content has the highest coefficient of variation (124.90%), followed by chlorophyll b content (118.49%) and chlorophyll a content (93.96%). The total chlorophyll content has the smallest coefficient of variation (85.88%). The content of chlorophyll a ranges from 0.0053 μg/mL to 0.9469 μg/mL, chlorophyll b varies from 0.0129 μg/mL to 0.7481 μg/mL, total chlorophyll ranges from 0.0372 μg/mL to 1.6950 μg/mL, and total anthocyanin ranges from 0.0037 U/g to 2.867 U/g. Correlation analysis reveals that chlorophyll a, chlorophyll b, and total chlorophyll content are significantly positively correlated with each other, but not significantly correlated with the five parameters measured by the colorimeter and total anthocyanin content (Figure S2). Total anthocyanin content is significantly negatively correlated with L* and *h*°, and significantly positively correlated with a* and *C* (Figure S2).

**Figure 2:**
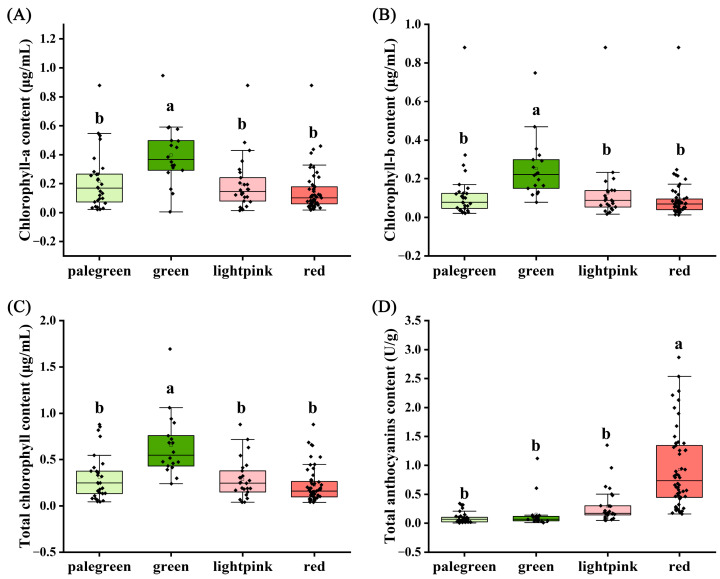
Comparison of chlorophyll and anthocyanin in cultivated caladium germplasm resources of different color groups. (**A**) Chlorophyll-a content. (**B**) Chlorophyll-b content. (**C**) Total chlorophyll content. (**D**) Total anthocyanins content. Groups exhibiting significant differences were denoted by distinct letters (a, b) at a significance level of *p* < 0.05.

Figure 2 presents a comparison of chlorophyll and anthocyanin in cultivated caladium leaves of different color groups. For chlorophyll a, chlorophyll b, and total chlorophyll content, the green group exhibits the highest content, with mean values of 0.40 μg/mL, 0.25 μg/mL, and 0.65 μg/mL, respectively (Figure 2A–C; Table S3). The palegreen and lightpink groups rank second, while the red group has the lowest content. For total anthocyanin content, the red group has the highest mean content (0.93 U/g), followed by the lightpink group (0.29 U/g), with the green (0.15 U/g) and palegreen groups (0.09 U/g) having low content (Figure 2D; Table S3). Multiple comparison analysis reveals that the green group has significantly higher chlorophyll a, chlorophyll b, and total chlorophyll content than the other color groups, while the red group has significantly higher total anthocyanin content than the other three color groups.

### 2.2. Association Analysis of EST-SSR with Chlorophyll and Anthocyanin Content

**Figure 3:**
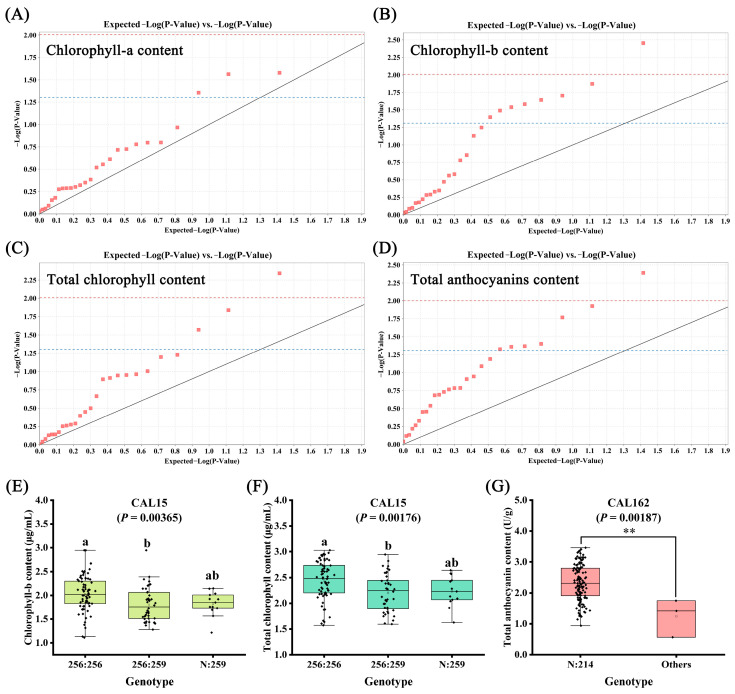
Association analysis of chlorophyll and anthocyanin based on 26 EST-SSRs. (**A**) QQ plot of chlorophyll-a content. (**B**) QQ plot of chlorophyll-b content. (**C**) QQ plot of total chlorophyll content. (**D**) QQ plot of total anthocyanins content. (**E**) Box chart analysis of chlorophyll-b content at CAL15 locus. (**F**) Box chart analysis of total chlorophyll content at CAL15 locus. (**G**) Box chart analysis of total anthocyanins content at CAL162 locus. Values above the blue dashed line indicate *p* < 0.05, and values above the red dashed line indicate *p* < 0.01. Groups exhibiting significant differences were denoted by distinct letters (a, b) at a significance level of *p* < 0.05. The “**” in (**G**) indicates *p* < 0.01.

Based on the SSR genotyping results from our previous study, an association analysis of chlorophyll a, chlorophyll b, total chlorophyll, and total anthocyanin content was conducted. The analysis revealed that at the *p* < 0.05 level, three, eight, three and seven loci were significantly associated with chlorophyll a, chlorophyll b, total chlorophyll, and total anthocyanin content, respectively, explaining 5.12% to 11.44% of the variation (Figure 3A–D; Table S4). At the *p* < 0.01 level, one locus each was identified to be highly significantly associated with chlorophyll b, total chlorophyll, and total anthocyanin content. Notably, CAL15 was highly significantly associated with both chlorophyll b and total chlorophyll, explaining 11.44% and 10.95% of the variation, respectively. In the CAL15 marker, individuals with the 256:256 genotype had mean chlorophyll b and total chlorophyll content of 2.03 μg/mL and 2.45 μg/mL, respectively, significantly higher than those with the 256:259 genotype (Figure 3E,F). Additionally, CAL162 was highly significantly associated with total anthocyanin content, explaining 8.48% of the variation. In the CAL162 marker, the genotype with the 214 fragment had a mean total anthocyanin content of 2.34 U/g, significantly higher than the mean total anthocyanin content in other genotypes (Figure 3G).

### 2.3. Anthocyanin Metabolomics Analysis of ‘Lieyanxiongxin’ and Its White Leaf Mutant

**Figure 4:**
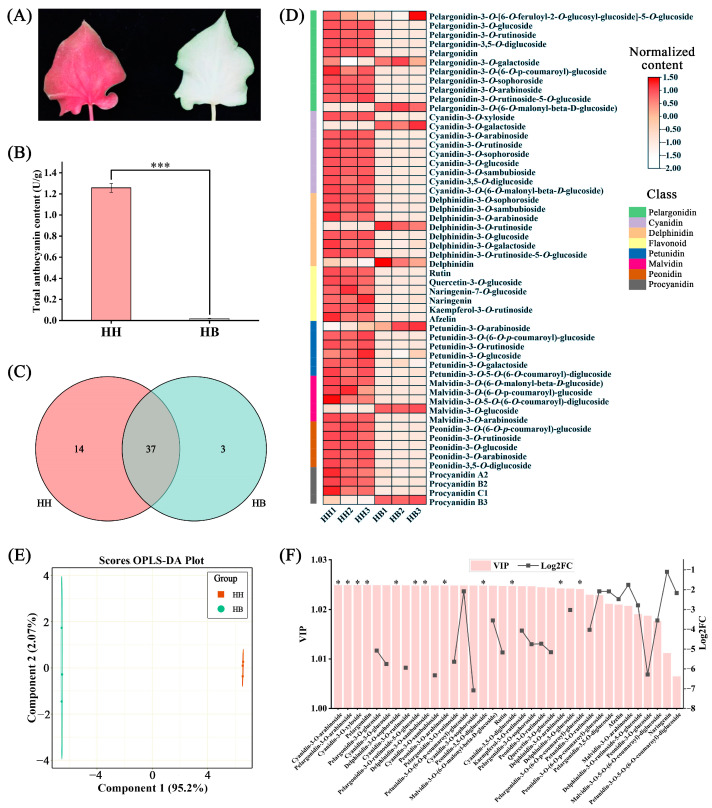
Anthocyanin metabolome analysis of ‘Lieyanxiongxin’ (HH) and its white-leaf mutant (HB). (**A**) Leaf images of red-leaf ‘Liyanxiongxin’ and its white-leaf mutant. (**B**) Comparison of total anthocyanin content between HH and HB. (**C**) Number of common and unique anthocyanin metabolites between HH and HB. (**D**) Heatmap. (**E**) OPLS-DA score plot. (**F**) VIP and log_2_FC values of significantly different anthocyanin metabolites between HH and HB leaves. The “***” in (**B**) indicates *p* < 0.001. The “*” in (**F**) indicates the anthocyanin metabolites unique to HH.

‘Liyanxiongxin’ (HH) is a caladium cultivar with red leaves. Interestingly, we found a white-leaf mutant (HB) of HH during its mass production. Except for the leaf color, HH and HB are almost identical in other traits (Figure 4A). The anthocyanin analysis of ‘Lieyanxiongxin’ and its white leaf mutant revealed that the total anthocyanin content of ‘Lieyanxiongxin’ leaves was 0.80 U/g, significantly higher than that of the white leaf mutant HB (0.05 U/g) (Figure 4B). To identify the anthocyanin types, we performed high performance liquid chromatography-mass spectrometry (HPLC-MS) analysis. Figures S3 and S4 show the total ion chromatogram and extracted ion chromatogram, respectively. The anthocyanin metabolomics analysis identified 54 anthocyanin compounds, including 11 pelargonidins, nine cyanidins, seven delphinidins, six flavonoids, six petunidins, five malvidins, five peonidins, and four procyanidins. Among these, HH had 14 unique metabolites, including Cyanidin-3-*O*-xyloside, cyanidin-3-*O*-arabinoside, cyanidin-3,5-*O*-diglucoside, delphinidin-3-*O*-sophoroside, delphinidin-3-*O*-sambubioside, delphinidin-3-*O*-arabinoside, malvidin-3-*O*-(6-*O*-malonyl-beta-*D*-glucoside), pelargonidin, pelargonidin-3-*O*-arabinoside, pelargonidin-3-*O*-(6-*O*-*p*-coumaroyl)-glucoside, pelargonidin-3-*O*-rutinoside-5-*O*-glucoside, peonidin-3-*O*-arabinoside, peonidin-3,5-*O*-diglucoside, and petunidin-3-*O*-(6-*O*-*p*-coumaroyl)-glucoside. In contrast, HB had only three unique metabolites, including cyanidin-3-*O*-galactoside, delphinidin-3-*O*-rutinoside, and pelargonidin-3-*O*-(6-*O*-malonyl-beta-*D*-glucoside), and they shared 37 metabolites (Figure 4C). Additionally, there were 14 unique anthocyanins in HH, but their concentrations were all below 0.45 μg/g, with pelargonidin having the highest concentration.

The heatmap results indicated that most of the anthocyanin content in HH was higher than that in HB (Figure 4D). Orthogonal projections to latent structures discriminant analysis (OPLS-DA) results demonstrated a significant difference in the anthocyanin content between HH and HB leaves (Figure 4E). Compared with HH, 36 anthocyanins in the mutant HB were significantly down-regulated (VIP > 1, Log_2_FC < −1) (Figure 4F). Moreover, nine anthocyanins had |Log2FC| greater than 5, namely cyanidin-3-*O*-rutinoside, cyanidin-3-*O*-sophoroside, cyanidin-3-*O*-glucoside, cyanidin-3-*O*-sambubioside, pelargonidin-3-*O*-glucoside, pelargonidin-3-*O*-rutinoside, peonidin-3-*O*-glucoside, rutin and quercetin-3-*O*-glucoside. Their concentrations in HH leaves were 126.61 μg/g, 6.71 μg/g, 46.52 μg/g, 7.92 μg/g, 6.20 μg/g, 15.22 μg/g, 0.12 μg/g, 486.90 μg/g, and 187.95 μg/g, respectively. These compounds may be responsible for the red color of HH leaves.

### 2.4. Transcriptome Analysis of ‘Lieyanxiongxin’ and Its White Leaf Mutant

Following the filtering of raw sequencing data, a total of 487,622,460 clean reads (73.13 Gb) from the transcriptome were obtained. The Q_20_ value of the six samples exceeded 97.85%, and the Q_30_ value was greater than 93.00%, with a GC percentage ranging from 49.08 to 50.64% (Table S5). We assembled a total of 176,869 unigenes (mean length 1381.22 bp) representing 303,585 transcripts (mean length 1118.72 bp) (Table S6). The highest number of transcripts fell within the 200 to 300 bp range, accounting for 19.33%, while the lowest number of transcripts fell within the 1900 to 2000 bp range (1.38%). The highest number of unigenes fell within the 300 to 400 bp range, accounting for 10.69% (Table S6).

**Figure 5:**
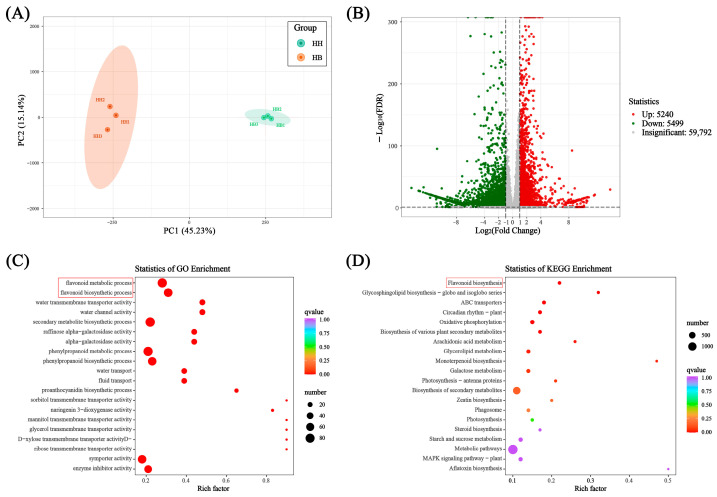
Transcriptome analysis of ‘Lieyanxiongxin’ (HH) and its white-leaf mutant (HB). (**A**) PCA score plot. (**B**) Volcano plot of differentially expressed genes. (**C**) GO enrichment analysis of differentially expressed genes. (**D**) KEGG enrichment analysis of differentially expressed genes. The items with red bracket are related to anthocyanins biosynthesis.

We annotated a total of 176,869 unigenes against the KEGG, NR, SwissProt, TrEMBL, KOG, GO, and Pfam databases (Table S7). Of these unigenes, 94,815 (53.61%) were annotated in at least one database. The number of annotated unigenes was highest in TrEMBL (92,695) and lowest in Pfam (54,796). Further BLAST searches against other databases showed that 67,138, 92,388, 58,515, 53,732, and 75,370 unigenes had matches in KEGG, Nr, SwissProt, KOG, and GO databases, respectively. Principal component analysis (PCA) and correlation analysis showed that the correlation between different duplicate samples was high, while the correlation between different processed samples was low (Figure 5A and Figure S5).

The differential expression genes (DEGs) were identified by DESeq based on the Fragments Per Kilobase of exon model per million mapped fragments (FPKM). DEGs with |log2 (fold change)| ≥ 1 and FDR < 0.05 were considered significantly different. Compared with the parent HH, there were 5240 genes significantly up-regulated and 5499 genes significantly down-regulated in HB (Figure 5B). GO enrichment analysis results showed that the most significant top two pathways were flavonoid metabolic process and flavonoid biosynthetic process (Figure 5C). KEGG enrichment analysis showed that flavonoid biosynthesis was the most significantly enriched pathway (Figure 5D).

### 2.5. Analysis of Anthocyanin Biosynthesis Pathway and Identification of Transcription Factors

**Figure 6:**
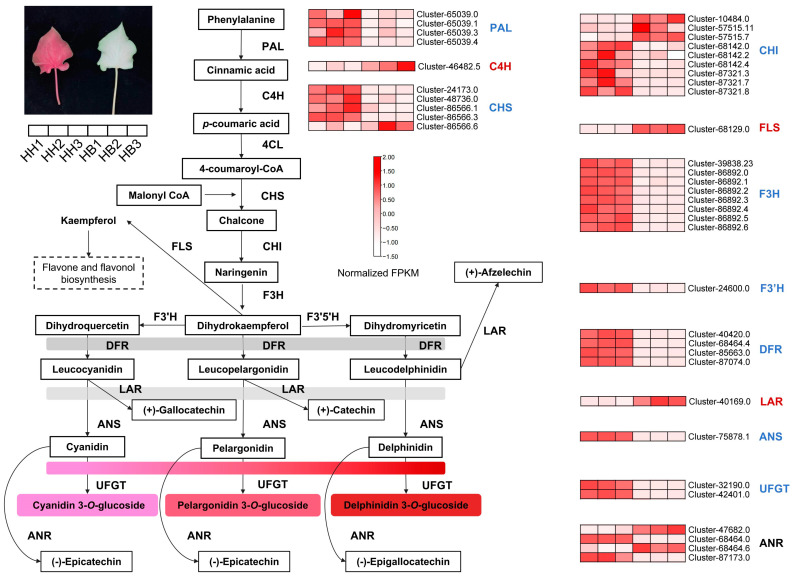
Differential expression analysis of anthocyanin biosynthesis pathway genes in ‘Lieyanxiongxin’ (HH) and its white-leaf mutant (HB). Red gene family names indicate that up-regulated genes are predominant. Blue gene family names indicate that down-regulated genes are dominant. Black gene family names indicate that the number of up-regulated and down-regulated genes are comparable. PAL: phenylalanine ammonia-lyase; C4H: cinnamate 4-hydroxylase; 4CL: 4-coumarate-CoA ligase; CHS: chalcone synthase; CHI: chalcone isomerase; F3H: flavonone 3-hydroxylase; F3′H: flavonoid 3′-hydroxylase; FLS: flavonol synthase; F3′5′H: flavonoid 3′5′-hydroxylase; DFR: dihydroflavonol reductase; LAR: leucoanthocyanidin reductase; ANS: anthocyanidin synthase; UFGT: UDP-glycose flavonoid glycosyltransferase. ANR: anthocyanidin reductase.

Metabolome data analysis showed that anthocyanin-related metabolites increased significantly. To further identify the cause of the loss of anthocyanin in HB leaves, we pinpointed 61 DEGs related to anthocyanin biosynthesis among the differentially expressed genes (Figure 6; Table S8). Anthocyanins belong to flavonoids, which share a common synthesis main chain with other flavonoids upstream and form various anthocyanins by branch synthesis reactions from dihydrokaempferol. Compared with the control HH, most of the *PAL*, *CHS*, *CHI*, *F3H*, *F3′H*, *DFR*, *ANS*, *UFGT*, and *ANR* genes that were positively correlated with anthocyanin accumulation in HB leaves were significantly down-regulated. At the same time, the *LAR* gene that promotes the synthesis of other substances from the anthocyanin precursor substances leucocyanidin, leucopelargonidin, and leucodelphinidin, respectively, was significantly up-regulated, and the *FLS* gene that catalyzes the formation of kaempferol from dihydrokaempferol was also significantly up-regulated. In addition, we also identified differentially expressed transcription factors.

**Figure 7:**
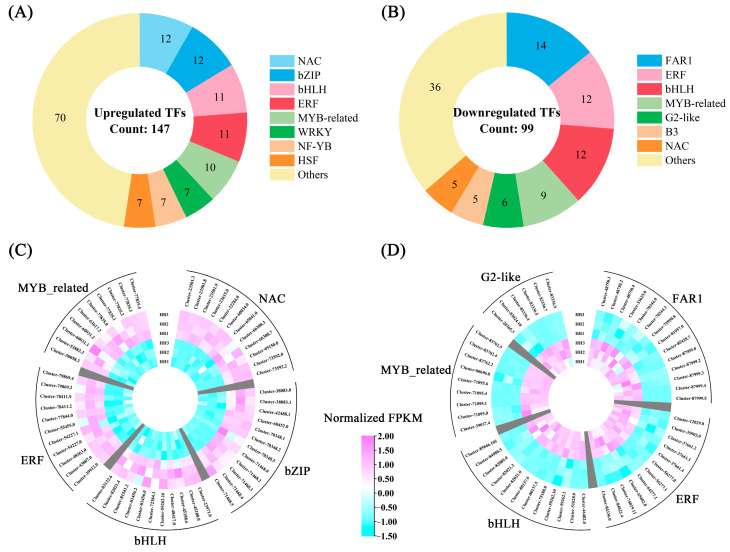
Differential expression analysis of transcription factors in ‘Lieyanxiongxin’ (HH) and its white-leaf mutant (HB). (**A**) Up-regulated differentially expressed transcription factors. (**B**) Down-regulated differentially expressed transcription factors. (**C**) Heatmap of gene expression of top five up-regulated transcription factor families. (**D**) Heatmap of gene expression of top five down-regulated transcription factor families.

In the transcriptome, we identified a total of 2523 transcription factors (Table S9). The ten most abundant families of these transcription factors, listed in order of their prevalence, were bHLH, C3H, ERF, FAR1, bZIP, MYB-related, C2H2, WRKY, G2-like, and NAC (Figure S6). Compared to the control parent HH, we identified 246 differentially expressed transcription factors in HB leaves. Among these, 147 transcription factors were up-regulated, including 12 *NAC*, 12 *bZIP*, 11 *bHLH*, 11 *ERF*, 10 *MYB*-related, seven *WRKY*, seven *NF-YB*, seven *HSF*, and 70 other transcription factors (Figure 7A; Table S10). Conversely, 99 transcription factors were down-regulated, including 14 *FAR1*, 12 *ERF*, 12 *bHLH*, nine *MYB*-related, six *G2-like*, five *B3*, five *NAC*, and 36 other transcription factors (Figure 7B; Table S11). The high number of up-regulated and down-regulated genes in the *bHLH*, *ERF*, and *MYB*_related families suggests that they may play a certain role in the formation of the HB white leaf trait. Figure 7C,D illustrate the gene expression heatmap of the top five gene families, ranked by the number of up-regulated and down-regulated transcription factors.

### 2.6. Quantitative Real-Time PCR (qRT-PCR) Analysis

**Figure 8:**
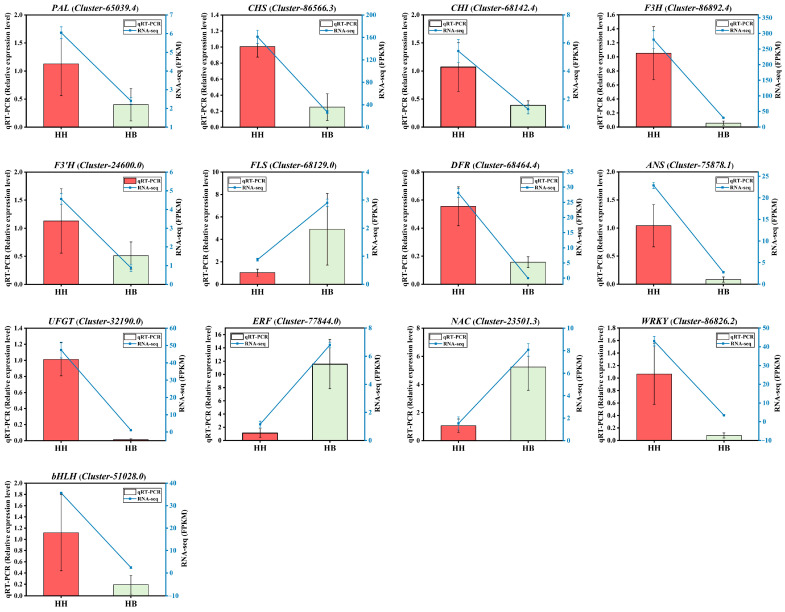
qRT-PCR verification of 13 key genes. HH stands for ‘Lieyanxiongxin’. HB stands for its white-leaf mutant.

Based on DEGs, 13 key genes were selected for qRT-PCR validation (Table S11). Nine of these genes were related to anthocyanin biosynthesis, including *PAL* (*Cluster-65039.4*), *CHS* (*Cluster-86566.3*), *CHI* (*Cluster-68142.4*), *F3H* (*Cluster-86892.4*), *F3′H* (*Cluster-24600.0*), *FLS* (*Cluster-68129.0*), *DFR* (*Cluster-68464.4*), *ANS* (*Cluster-75878.1*), and *UFGT* (*Cluster-32190.0*). The other four genes were transcription factors, namely *ERF* (*Cluster-77844.0*), *NAC* (*Cluster-23501.3*), *WRKY* (*Cluster-86826.2*), and *bHLH* (*Cluster-51028.0*). The qRT-PCR results agreed with the FPKM results, confirming the reliability and reproducibility of the RNA-seq data (Figure 8).

## 3. Discussion

In our previous study, we classified 144 cultivated caladium germplasm resources into four color groups based on colorimeter measurement and multivariate statistical analysis [14]. In this study, we further analyzed the differences in chlorophyll and anthocyanin across different color groups, finding significant pigment differences among the groups. Correlation analysis revealed that total anthocyanin content was significantly positively correlated with a* and *C*, and significantly negatively correlated with L* and *h*°. This aligns with previous studies on flower color in *Gerbera hybrida* Lynch [20], potted multiflora chrysanthemum (*Chrysanthemum morifolium* Ramat.) [21], *Gloriosa superba* Linnaeus [22], and other plants, where the a* value was significantly positively correlated with anthocyanin content. This suggests that colorimeter measurement can be used to predict the anthocyanin content of cultivated caladium leaves, providing an efficient method for rapid detection of cultivated caladium leaf anthocyanin.

Association analysis is a crucial method for mining molecular markers associated with traits. In this study, we associated three, eight, three, and seven loci with chlorophyll a, chlorophyll b, total chlorophyll, and total anthocyanin content, respectively, using the MLM model. Previous studies have successfully identified loci significantly associated with chlorophyll or anthocyanin in the leaves or petals of various plants, such as tea plant (*Camellia* spp. Kamel) [23], peanut (*Arachis hypogaea* L.) [24], rose (*Rosa rugosa* Thunb.) [25], *Gerbera hybrida* [20], rice (*Oryza sativa* L.) [26,27,28], and *Perilla frutescens* (L.) Britt. [29], through association analysis. Chlorophyll and anthocyanin content are both determined by multiple genes [30]. Therefore, multiple loci associated with chlorophyll or anthocyanin content can be mined in both this study and previous studies. Moreover, when performing association analysis of these complex quantitative traits in hybrid plants, the phenotypic explanatory rate of the associated loci is often relatively low [20,31]. In this study, the significant loci identified explained 5.12% to 11.44% of the phenotypic variation, respectively, and were significantly correlated with chlorophyll a, chlorophyll b, total chlorophyll, and total anthocyanin content. Due to the lack of relevant whole genome sequencing information, these significantly associated EST-SSR markers can provide reference for the genetic variation and molecular marker-assisted selective breeding of cultivated caladium leaf pigment.

Anthocyanin metabolic disorder is a primary factor contributing to the lighter leaf color in plants. In this study, we initially identified 36 significantly down-regulated anthocyanin metabolites through metabolomics analysis. Subsequently, transcriptomics revealed several key genes of anthocyanin biosynthesis that were significantly down-regulated. Concurrently, the *LAR* and *FLS* genes, which promote the synthesis of other substances from anthocyanin precursor substances, were significantly up-regulated. This combination of factors led to the white leaf mutation of ‘Lieyanxiongxin’. Previous studies have demonstrated that altering the structure or expression of *CHS*, the key enzyme gene in the first step of flavonoid biosynthesis, can result in white flowers in petunia [32,33]. Similarly, inhibiting the expression of *CHS* and *ANS* can produce white gentian flowers [34]. Post-transcriptional gene silencing of *CHS* is also a key factor in the formation of white petals in *Dahlia variabilis* Desh. [35]. In *Nelumbo nucifera* Gaertn., the low expression of the *UFGT* gene is a significant factor in the appearance of white petals [36]. In our study, several down-regulated genes in the flavonoid biosynthesis pathway were identified in HB, including four *CHS*, one *ANS*, and two *UFGT*, as well as several *PAL*, *CHI*, *F3H*, *F3′H*, and *DFR*. That is, except for *C4H*, other synthesis enzyme genes that are conducive to anthocyanin accumulation were significantly down-regulated, resulting in the loss of red color in the leaves.

**Figure 9:**
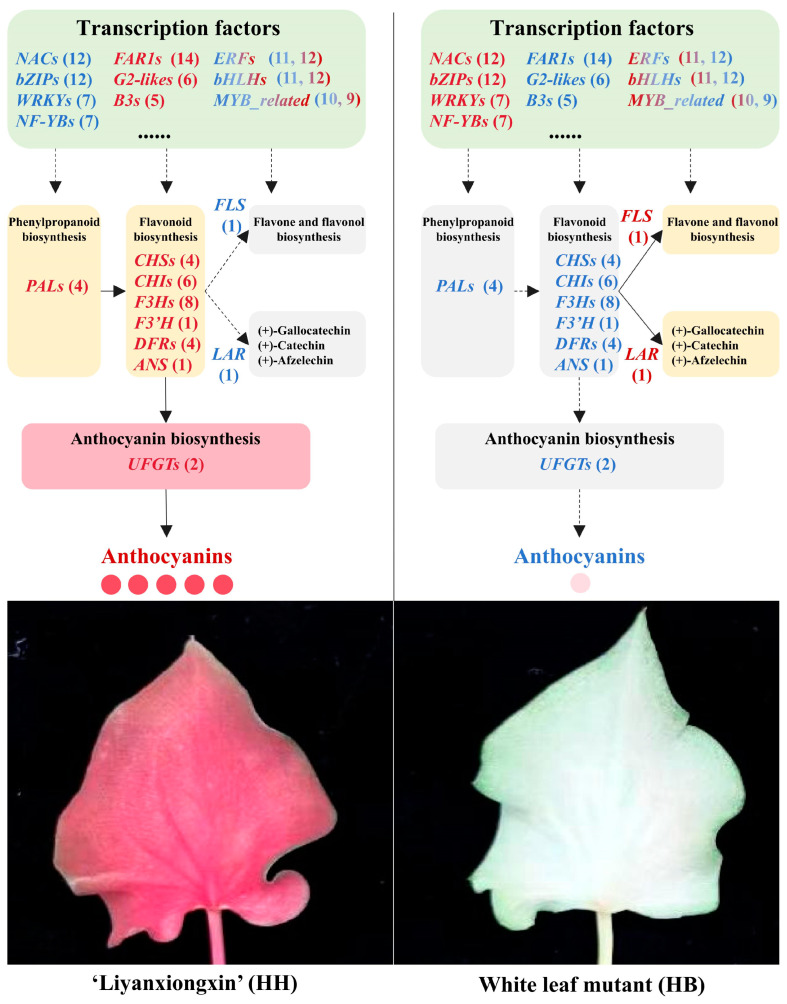
Schematic diagram of the potential metabolic and molecular regulation of the loss of red color in the leaves of the white-leaf mutant of ‘Lieyanxiongxin’. Red font indicates higher gene expression or metabolite content. Blue font indicates lower gene expression or metabolite content. Red-blue gradient of *ERF*, *bHLH* and *MYB_related* indicates a large number of both up-regulated and down-regulated genes in these gene families. The red numbers indicate the number of up-regulated genes, and the blue numbers indicate the number of down-regulated genes. The information on these key differentially expressed genes is listed in Tables S8, S10 and S11.

Transcription factors such as MYB have been proven to regulate anthocyanin biosynthesis in plant organs [37,38]. Additionally, bHLH, NAC, bZIP, ERF, and other transcription factors can either positively or negatively regulate anthocyanin biosynthesis [39,40]. Interestingly, some transcription factors can even regulate the appearance of the white phenotype in plant tissues. For example, PbMYB26 can degrade anthocyanin in the petals, leaves, and pedicels of pear by activating the expression of *PbLAC4*, and even produce a white phenotype [41]. In tobacco, CRISPR/Cas9-induced mutation in the *RcMYB3* gene of *Rubus chingii* Hu. leads to the appearance of white petals [42]. In *Syringa oblata* Lindl., ERF plays an important role in the color transition period (from purple to light purple near white), while the MBW complex participates in the whole process of color change [43]. This study detected various transcription factors (TFs) that were differentially expressed between the red (HH) and white (HB) leaves of cultivated caladium. Among the up-regulated TFs, 12 *NAC*, 12 *bZIP*, 11 *bHLH*, 11 *ERF*, 10 MYB-related, and others were found. Among the down-regulated TFs, 14 *FAR1*, 12 *ERF*, 12 *bHLH*, nine *MYB*-related, six *G2-like*, and others were found. These differentially expressed TFs may play important roles in regulating anthocyanin accumulation in caladium leaves, and their functions need to be validated by further molecular biology experiments. Interestingly, besides the commonly reported TFs, 14 *FAR1* that were significantly down-regulated in the white leaf mutant were also detected. *FAR1* transcription factors play an important role in light signal transduction and plant growth and development [44], but there is no report on their regulation of anthocyanin biosynthesis, which suggests that the abnormality of the light signal pathway in the white leaf mutant HB may lead to the obstruction of anthocyanin synthesis. A possible metabolic and molecular regulatory schematic diagram of the loss of red color in the leaves of ‘Lieyanxiongxin’ white-leaf mutant is shown in Figure 9. The regulatory mechanism of these TFs on anthocyanin synthesis in cultivated caladium leaves remains to be further clarified. Moreover, it is worth exploring whether this white leaf mutation mechanism has a similar effect in other cultivars.

## 4. Materials and Methods

### 4.1. Plant Materials

A total of 144 cultivated caladium germplasm resources were from China (42), Thailand (52), and the United States (50) and cultivated in a greenhouse of Baiyun Base of Guangdong Academy of Agricultural Sciences (23.40° N, 113.45° E). The greenhouse conditions were 28 ± 2 °C and 75–80% humidity. We extracted chlorophyll from 126 germplasms and total anthocyanin from 136 germplasms. Some germplasms had insufficient quantity for extraction. Table S1 lists the details of each germplasm. A red-leaf caladium variety, ‘Lieyanxiongxin’ (HH), produced a white-leaf mutant (HB) during mass tissue culture. HB was then selected and propagated vegetatively. Apart from the leaf color, HH and HB share almost the same traits (Figure 4A and Figure S1).

### 4.2. Chlorophyll Measurement

Chlorophyll content was measured following the method of Arnon [45] with slight modifications. A mixture of ethanol, acetone, and water (4.5:4.5:1 *v*/*v*/*v*) was used to extract chlorophyll from 0.2 g of fresh leaf. The mixture was stored at approximately 4 °C in the dark for 24 h. A UV-1200 spectrophotometer (MAPADA, Shanghai, China) was used to measure the absorbance at wavelengths of 665 and 649 nm. We calculated the concentrations (μg/mL) as follows: Chlorophyl-a content = 13.95 × A_665_ − 6.88 × A_649_; Chlorophyl-b content = 24.96 × A_649_ − 7.32 × A_665_. Total chlorophyll content = 6.63 × A_665_ + 18.08 × A_649_. Each experiment was performed in triplicate.

### 4.3. Total Anthocyanin Measurement

The method of Zhou et al. [20] was adapted to measure the total anthocyanin content. Fresh leaf (0.5 g) was ground into powder with liquid nitrogen and added to a 10 mL extraction solution of HCl and methanol (1:99 *v*/*v*). The solution was stored at 4 °C for 24 h until the leaf tissue color faded. The solution was filtered with filter paper, and the filtrate was collected. A UV-1200 spectrophotometer (MAPADA, Shanghai, China) was used to measure the absorbance at wavelengths of 530 nm and 657 nm. We calculated the anthocyanin content (U/g) by the formula total anthocyanin = A_530_ − 0.25 × A_657_. We performed each experiment in three replicates.

### 4.4. Anthocyanin Metabolomics Analysis

HPLC-grade methanol (MeOH) from Merck (Darmstadt, Germany) was used with MilliQ water (Millipore, Burlington, NJ, USA) for all experiments. All standards were purchased from isoReag (Shanghai, China). Formic acid and hydrochloric acid were acquired from Sigma-Aldrich (St. Louis, MO, USA) and Xinyang Chemical Reagent (Ningxiang, China), respectively. Stock solutions of standards (1 mg/mL in 50% MeOH) were prepared and stored at –20 °C. We diluted the stock solutions with 50% MeOH to obtain the working solutions before analysis. We freeze-dried the sample, ground it (30 Hz, 1.5 min), and preserved it at –80 °C until use. We weighed 50 mg of powder and extracted it with 0.5 mL of methanol/water/hydrochloric acid (500:500:1, *v*/*v*/*v*). The extract was vortexed for 5 min, sonicated for 5 min, and centrifuged at 12,000× *g* at 4 °C for 3 min. The extraction was repeated with the residue under the same conditions. The supernatants were collected and filtered through a 0.22 μm membrane filter (Anpel) (Millipore, Burlington, NJ, USA) before LC–MS/MS analysis.

The sample extracts were analyzed using a UPLC–ESI–MS/MS system (UPLC ExionLC™ AD; MSˈ Applied Biosystems 6500 Triple Quadrupole, AB Sciex, Singapore). The analytical conditions were as follows: UPLC: column, Waters ACQUITY BEH C18 (1.7 µm, 2.1 mm × 100 mm); solvent system, water (0.1% formic acid): methanol (0.1% formic acid); gradient program, 95:5 *v*/*v* at 0 min, 50:50 *v*/*v* at 6 min, 5:95 *v*/*v* at 12 min, hold for 2 min, 95:5 *v*/*v* at 14 min; hold for 2 min; flow rate, 0.35 mL/min; temperature, 40 °C; injection volume, 2 μL. We used a triple quadrupole-linear ion trap mass spectrometer (QTRAP), QTRAP^®^ 6500+ LC–MS/MS System (AB Sciex, Singapore), with an ESI Turbo Ion-Spray interface, in positive ion mode and controlled by Analyst 1.6.3 software (Sciex) to acquire linear ion trap (LIT) and triple quadrupole (QQQ) scans. The ESI source operation parameters were as follows: ion source, ESI+; source temperature 550 °C; ion spray voltage (IS) 5500 V; curtain gas (CUR) was set at 35 psi. Scheduled multiple reaction monitoring (MRM) was used to analyze anthocyanins. We acquired data using Analyst 1.6.3 software (Sciex). We carried out three replicates for each experiment.

The metabolites detected by mass spectrometry were identified using the MWDB (Metware Database, www.metware.cn, accessed on 20 December 2022) database based on the standards. All metabolites were quantified using Multiquant 3.0.3 software (Sciex). We adjusted the mass spectrometer parameters, including the declustering potentials (DP) and collision energies (CE) for individual MRM transitions. A specific set of MRM transitions was monitored for each period according to the metabolites eluted within this period. The content of metabolites (μg/g) in the sample was calculated by the formula: content = cV/1,000,000/m. c: the concentration value (ng/mL) obtained by substituting the peak area of the sample into the standard curve; V: the volume (μL) of the extraction solution; m: the mass (g) of the sample. All anthocyanins had standard curves, which are shown in Table S12. We identified the significantly regulated metabolites between groups by absolute |Log_2_FC (fold change)| > 1 and VIP (VIP > 1).

### 4.5. Transcriptome Analysis

#### 4.5.1. RNA Extraction, Quantification and Qualification

We conducted RNA-seq analysis on leaves with three biological replicates for each period, resulting in six samples. We submitted the samples to Novogene Bioinformatics Technology Co., Ltd. (Beijing, China) for RNA sequencing. We examined RNA degradation and contamination on 1% agarose gels. We verified RNA purity using the NanoPhotometer^®^ spectrophotometer (IMPLEN, Westlake Village, CA, USA). We determined RNA concentration using Qubit^®^ RNA Assay Kit in Qubit^®^2.0 Flurometer (Life Technologies, Carlsbad, CA, USA). We evaluated RNA integrity using the RNA Nano 6000 Assay Kit of the Bioanalyzer 2100 system (Agilent Technologies, Santa Clara, CA, USA).

#### 4.5.2. Transcriptome Sequencing

The RNA samples were prepared using 1 µg of RNA per sample as input material. We generated sequencing libraries and attributed sequences to each sample using NEBNext^®^ UltraTM RNA Library Prep Kit for Illumina^®^ (NEB, Ipswich, MA, USA) and index codes. Briefly, mRNA was isolated from total RNA using poly-T oligo-attached magnetic beads. Divalent cations and high temperature in NEBNext First Strand Synthesis Reaction Buffer (5×) (NEB, Ipswich, MA, USA) were used to cleave the mRNA. Random hexamer primer and M-MuLV Reverse Transcriptase (RNase H-) were used to synthesize the first strand cDNA. DNA Polymerase I and RNase H were used to synthesize the second strand cDNA. Exonuclease/polymerase activities were used to blunt the remaining overhangs. Adenine was added to the 3′ ends of DNA fragments and ligated NEBNext Adaptor with hairpin loop structure for hybridization. cDNA fragments of 250~300 bp in length were selected by purifying the library fragments with AMPure XP system (Beckman Coulter, Beverly, CA, USA). Size-selected, adaptor-ligated cDNA was treated with 3 µL of USER Enzyme (NEB, Ipswich, USA) at 37 °C for 15 min and 95 °C for 5 min before PCR. We performed PCR with Phusion High-Fidelity DNA polymerase, Universal PCR primers and Index (X) Primer. We purified the PCR products (AMPure XP system) and checked the library quality on the Agilent Bioanalyzer 2100 system. The index-coded samples were clustered on a cBot Cluster Generation System using TruSeq PE Cluster Kit v3-cBot-HS (Illumina). After cluster generation, the library preparations were sequenced on an Illumina platform and produced 150 bp paired-end reads.

#### 4.5.3. Assembly and Gene Annotation

Raw data were filtered using fastp, and reads removed with adapters, more than 10% N bases, or more than 50% low-quality (Q ≤ 20) bases. We used clean reads for all subsequent analyses. The transcriptome was assembled using Trinity [46] and related transcripts clustered into ‘gene’ clusters using Corset. Candidate coding regions within transcript sequences were identified using TransDecoder (v5.7.0). Gene function was annotated based on the following databases using diamond or HMMER: Nr, Swiss-Prot, Trembl, KEGG, GO, KOG/COG, and Pfam. Gene expression levels were estimated by RSEM, and the FPKM of each gene calculated based on the gene length. FPKM is a common method to estimate gene expression levels. Differential expression between the two groups was analyzed using DESeq2 (v1.42.0) [47]. The *p* value was corrected using the Benjamini and Hochberg method. We used the corrected *p* value and |log2foldchange| as the threshold for significant differential expression. Enrichment analysis was performed based on the hypergeometric test. We used the pathway unit for KEGG and the GO term for GO. We performed TF analysis of differentially expressed genes using iTAK (v1.6) [48].

### 4.6. qRT-PCR Analysis

The RNA-seq data were validated by quantitative real-time PCR (qRT-PCR) following standard procedures. Total RNA was isolated with a HiPure Plant RNA Mini Kit (Magen, Guangzhou, China), and its concentration was measured by a spectrophotometer according to the manufacturer’s recommendations. cDNA was synthesized from total RNA using the PrimeScript RT Reagent Kit (TaKaRa, Kusatsu, Japan) as per the manufacturer’s directions. About 1 µg of total RNA was reverse transcribed with the PrimeScriptTM RT reagent kit. qRT-PCR analysis was performed with the QTOWER3 instrument (Analytik Jena, Jena, Germany). Each qPCR reaction consisted of 20 µL final volume, containing 10 µL SYBR Green PCR Master Mix (Applied Biosystems), 0.5 µL of each primer (10 µM), 1 µL of cDNA template, and 8 µL of nuclease-free water. The thermal cycling profile was as follows: initial denaturation at 95 °C for 10 min, followed by 40 cycles of 95 °C for 15 s, 60 °C for 30 s, and 72 °C for 30 s. Fluorescence was measured at the end of each cycle. The qPCR experiments were performed in triplicate, and the data were analyzed using qPCRsoft (v3.1) software (Analytik Jena). The relative expression levels of the target genes were determined using the 2−^ΔΔCt^ method [49]. The primers of 13 key genes and reference gene are listed in Table S13.

### 4.7. Statistical Analysis

All analyses were performed using R packages unless otherwise stated [50]. The Shannon–Wiener index and coefficient of variation (CV) were calculated using the “vegan” package [51]. Pearson correlation analysis was performed and visualized using the built-in function of R language and the “corrplot” package (v0.92) [52]. Student’s t test and a one-way analysis of variance (ANOVA) were performed using the built-in function of R language. The mean values were compared using Duncan’s multiple range tests at a significance level of *p* < 0.05. Association analysis between traits and markers was performed using Tassel 4.3.6 software [53]. The association was considered significant when *p* < 0.05 and highly significant when *p* < 0.01. OPLS-DA analysis and VIP value calculation were performed using the “ropls” package (v1.34.0) [54]. Principal component analysis (PCA) was conducted using the “mixOmics” package (v6.0.0) [55].

## 5. Conclusions

In this study, we elucidated the diversity and mechanism of leaf pigments in cultivated caladium, a popular ornamental plant with various leaf colors. Chlorophyll and anthocyanin content of 137 cultivated caladium germplasm resources were measured, and significant differences among palegreen, green, lightpink and red leaf color groups were found. Three, eight, three, and seven EST-SSR loci were identified as significantly associated with chlorophyll a, chlorophyll b, total chlorophyll, and total anthocyanin content, respectively, explaining 5.12% to 11.44% of the phenotypic variation. The white leaf mutation of ‘Lieyanxiongxin’ was primarily due to the reduced content of nine anthocyanins, namely cyanidin-3-*O*-rutinoside, cyanidin-3-*O*-sophoroside, cyanidin-3-*O*-glucoside, cyanidin-3-*O*-sambubioside, pelargonidin-3-*O*-glucoside, pelargonidin-3-*O*-rutinoside, peonidin-3-*O*-glucoside, rutin, and quercetin-3-*O*-glucoside. This reduction in anthocyanin content was caused by the decreased expression of key genes in the anthocyanin biosynthesis pathway. The white leaf mutation might be caused by the disruption of anthocyanin biosynthesis and regulation, involving multiple enzyme genes (four *PAL*, four *CHS*, six *CHI*, eight *F3H*, one *F3′H*, one *FLS*, one *LAR*, four *DFR*, one *ANS* and two *UFGT*) and transcription factors (12 *NAC*, 12 *bZIP*, 23 *ERF*, 23 *bHLH*, 19 *MYB*_related, etc.). This study provided insights into the physiological and molecular basis of leaf color formation and variation in cultivated caladium, and could facilitate the selection and breeding of cultivated caladium leaf color.

## Data Availability

The transcriptome raw data have been submitted to the SRA database of the NCBI (PRJNA1055588).

## Supplementary Materials

The following supporting information can be downloaded at: https://www.mdpi.com/article/10.3390/ijms25010605/s1.
