# Supplementary figures and images for: Pigment Diversity in Leaves of Caladium × hortulanum Birdsey and Transcriptomic and Metabolic Comparisons between Red and White Leaves
Source: Int J Mol Sci. 2024 Jan 3;25(1):605. doi: 10.3390/ijms25010605 (PMC10779550; doi:10.3390/ijms25010605)

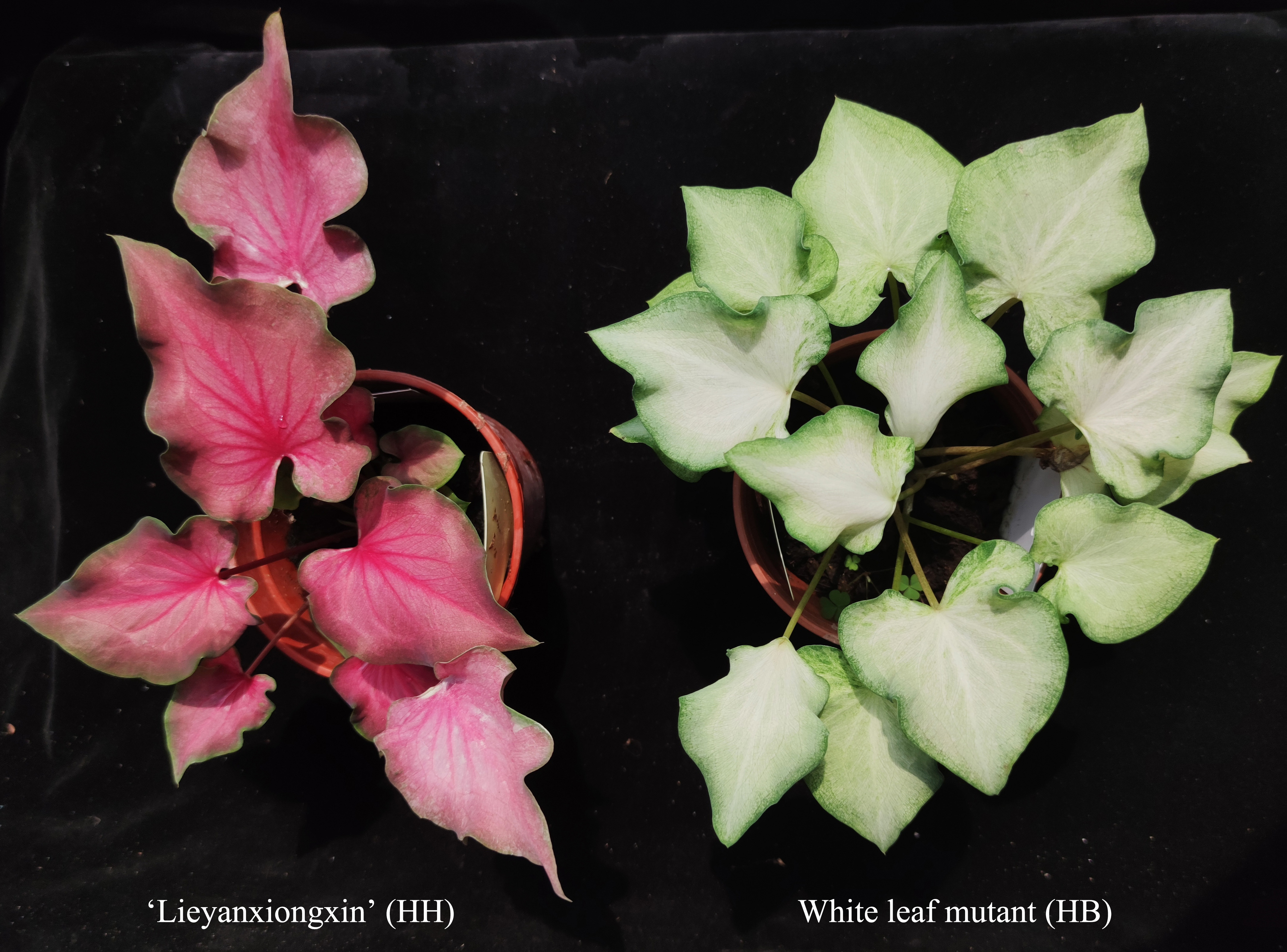

Supplement: Supplementary file 1 [file ijms-25-00605-s001.zip › Supplementary Files R2/Figure S1.jpg]

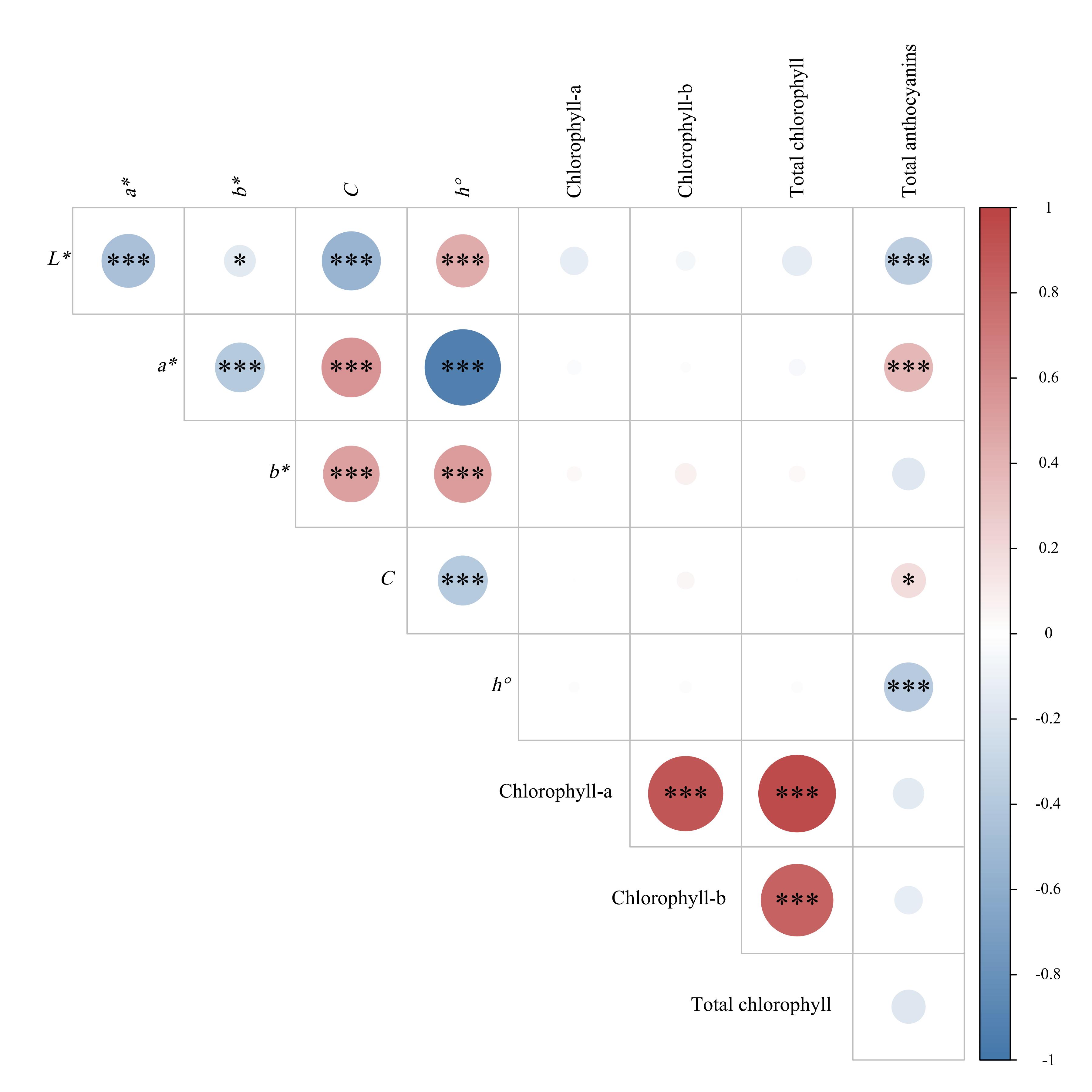

Supplement: Supplementary file 1 [file ijms-25-00605-s001.zip › Supplementary Files R2/Figure S2.jpg]

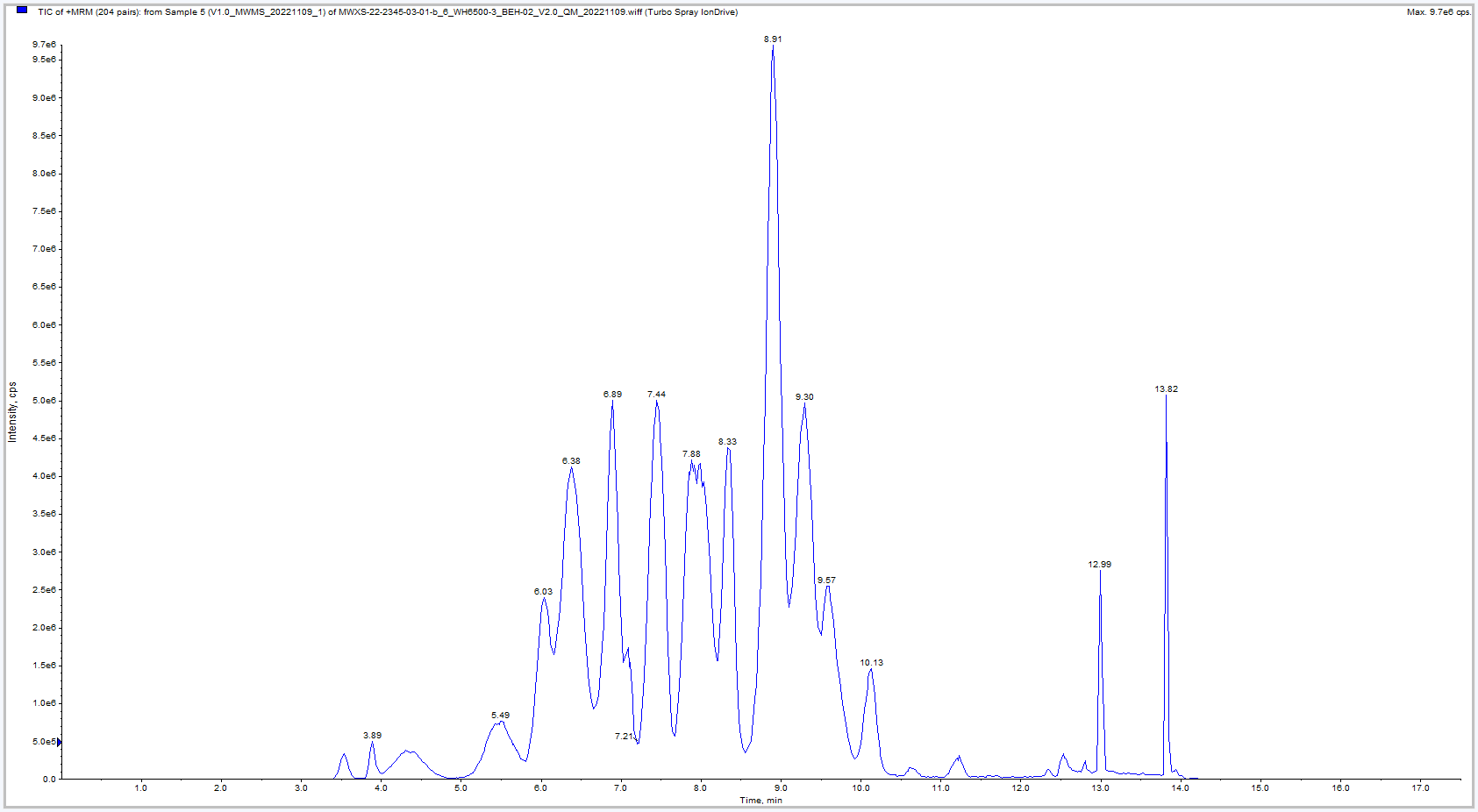

Supplement: Supplementary file 1 [file ijms-25-00605-s001.zip › Supplementary Files R2/Figure S3.png]

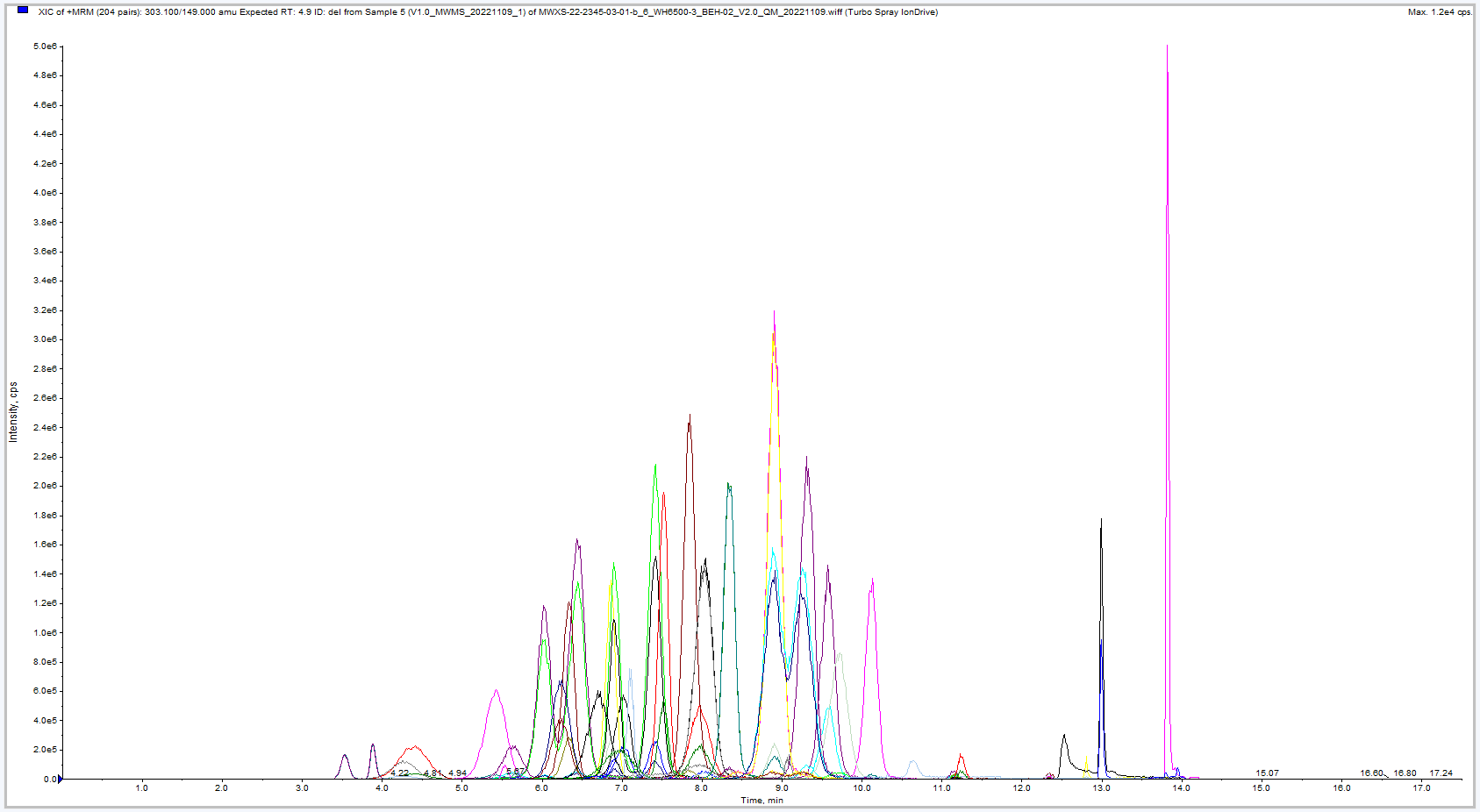

Supplement: Supplementary file 1 [file ijms-25-00605-s001.zip › Supplementary Files R2/Figure S4.png]

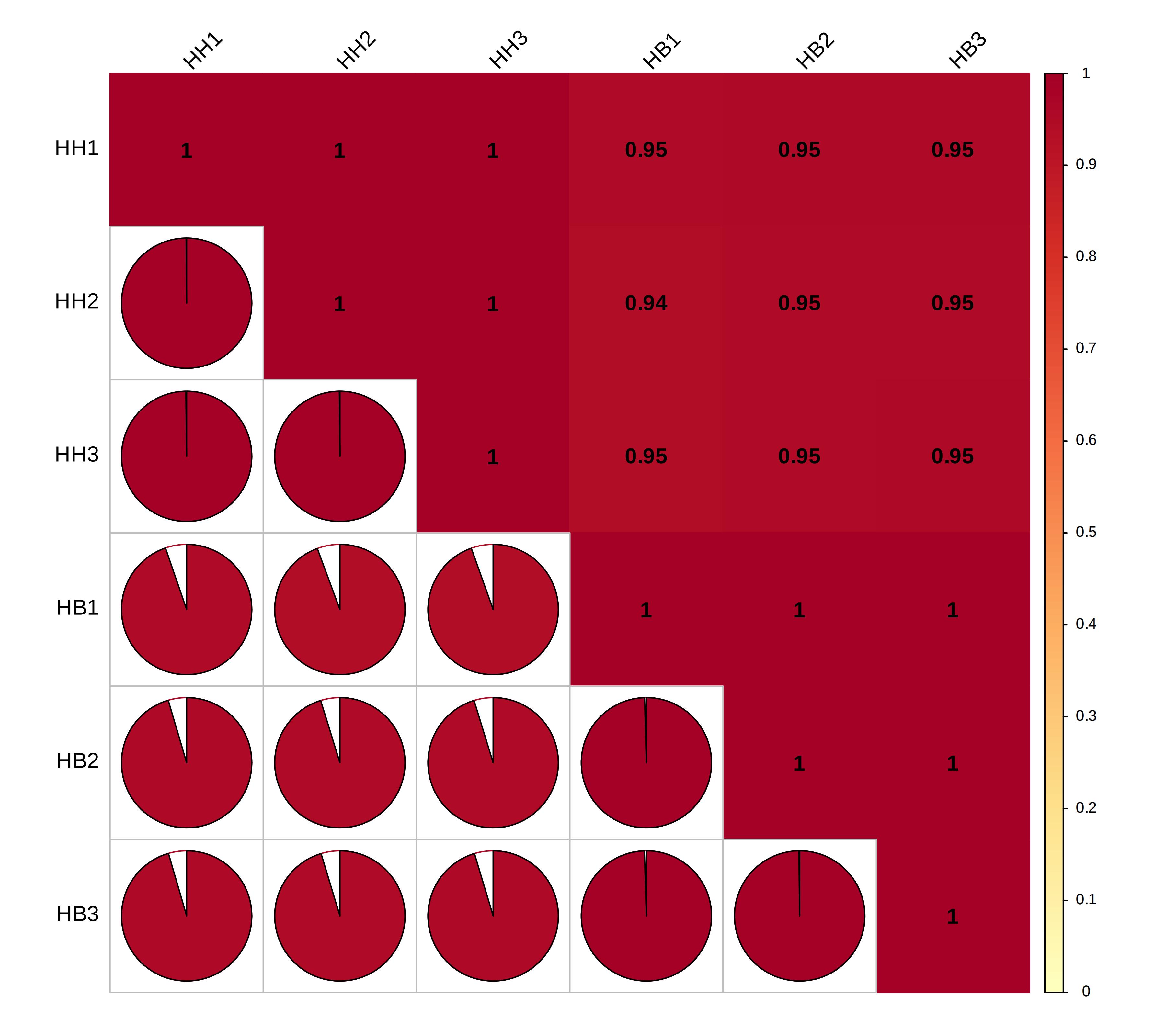

Supplement: Supplementary file 1 [file ijms-25-00605-s001.zip › Supplementary Files R2/Figure S5.jpg]

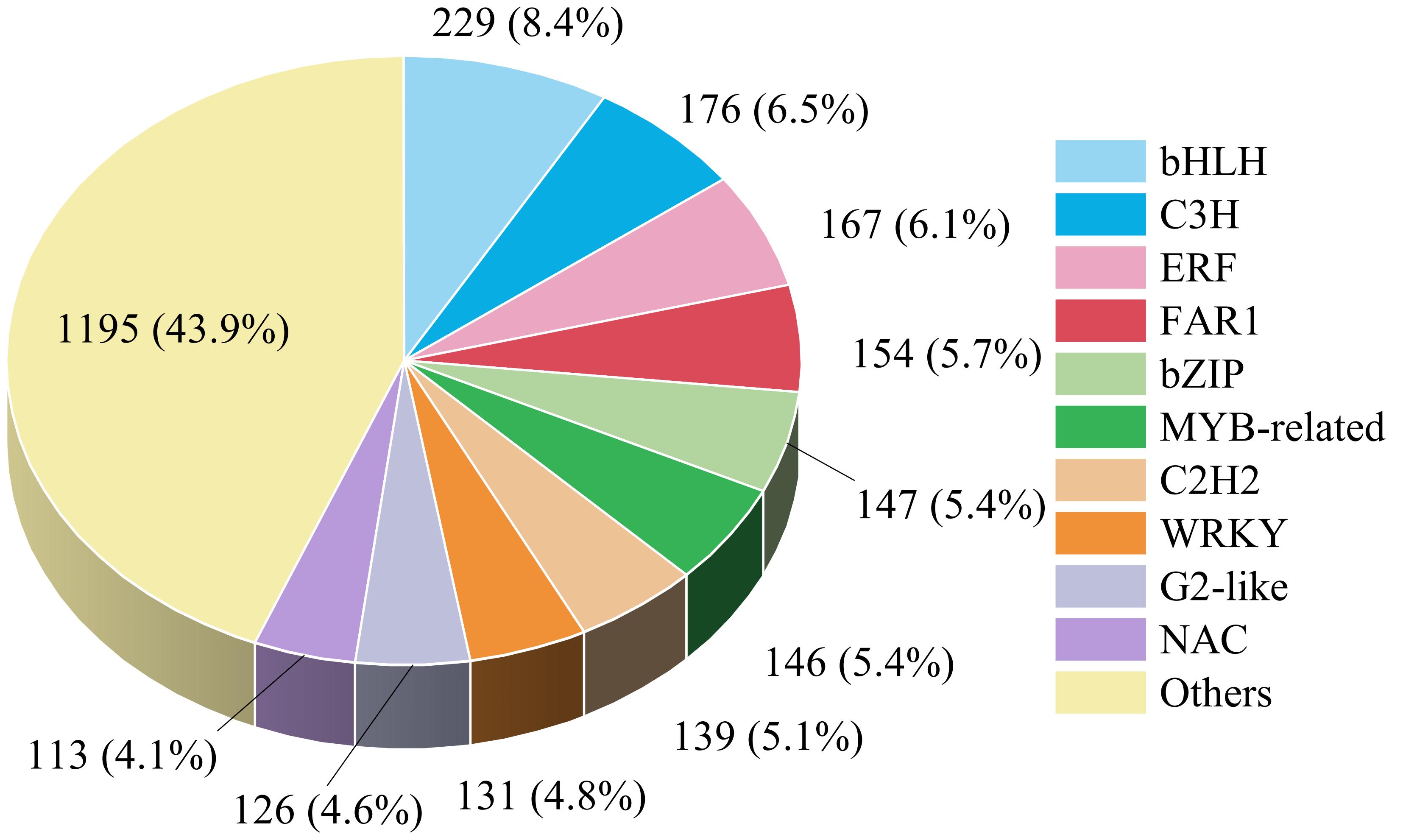

Supplement: Supplementary file 1 [file ijms-25-00605-s001.zip › Supplementary Files R2/Figure S6.jpg]
